# Hydrogen Embrittlement Detection Technology Using Nondestructive Testing for Realizing a Hydrogen Society

**DOI:** 10.3390/ma17174237

**Published:** 2024-08-27

**Authors:** Yamato Abiru, Hiroshi Nishiguchi, Masato Maekawa, Takara Nagata, Toshiya Itaya, Michie Koga, Toshiomi Nishi

**Affiliations:** 1 National Institute of Technology, Sasebo College, Nagasaki 857-1193, Japan; me2302@st.sasebo.ac.jp; 2 Department of Electrical and Electronic Information Engineering, Toyohashi University of Technology, Toyohashi 441-8580, Japan; maekawa.masato.cv@tut.jp; 3 Department of Computer Science and Engineering, Toyohashi University of Technology, Toyohashi 441-8580, Japan; nagata.takara.ul@tut.jp; 4 National Institute of Technology, Suzuka College, Mie 510-0294, Japan; itaya@info.suzuka-ct.ac.jp (T.I.);; 5 Kyuken Co., Ltd., Saga 849-0932, Japan; kogam@kk-kyuken.jp

**Keywords:** hydrogen embrittlement, nondestructive testing, eddy current testing, hammering testing

## Abstract

Crack detection in high-pressure hydrogen gas components, such as pipes, is crucial for ensuring the safety and reliability of hydrogen infrastructure. This study conducts the nondestructive testing of crack propagation in steel piping under cyclic compressive loads in the presence of hydrogen in the material. The specimens were hydrogen-precharged through immersion in a 20 mass% ammonium thiocyanate solution at 40 °C for 72 h. The crack growth rate in hydrogen-precharged specimens was approximately 10 times faster than that in uncharged specimens, with cracks propagating from the inner to outer surfaces of the pipe. The fracture surface morphology differed significantly, with flat surfaces in hydrogen-precharged materials and convex or concave surfaces in uncharged materials. Eddy current and hammering tests revealed differences in the presence of large cracks between the two materials. By contrast, hammering tests revealed differences in the presence of a half size crack between the two materials. These findings highlight the effect of hydrogen precharging on crack propagation in steel piping and underscore the importance of early detection methods.

## 1. Introduction

To solve environmental problems such as global warming and fossil fuel depletion, most countries worldwide are promoting a decarbonized society. To reduce greenhouse gas emissions such as carbon dioxide to zero or achieve carbon neutrality, the use of hydrogen energy, a clean and renewable energy source, is attracting considerable attention. In many countries, studies have focused on fuel-cell vehicles (FCVs) and hydrogen stations [1,2,3].

Solving the hydrogen embrittlement problem is critical for promoting hydrogen utilizations [4,5,6,7,8,9,10,11,12,13,14,15,16,17,18,19,20,21,22,23,24,25,26,27,28,29,30,31,32,33,34,35,36,37,38]. In this case, hydrogen penetrates the metallic materials used in a hydrogen environment and reduces material strength properties. Japan Automobile Research Institute (JARI) S-001 [39] has selected austenitic stainless steel SUS316L and aluminum alloy A6061-T6 as materials for use in FCVs. However, austenitic stainless steel SUS316L has many alloying elements such as chromium Cr, Ni, and molybdenum Mo, which increase the cost of this material. Thus, the products used in a hydrogen environment are expensive and the realization of a hydrogen energy society becomes difficult. To realize a hydrogen energy society, the cost of the products used in a hydrogen environment should be reduced. However, carbon steel and low-alloy steel cannot be used because they are classified as severely or extremely embrittled by NASA’s tensile tests in hydrogen gas [23]. However, the use of such low-cost materials based on experimental and analytical facts is being considered for use in a safe condition.

Studies have focused on investigating the fatigue crack propagation characteristics in austenitic stainless steels such as SUS304 and SUS316L, carbon steels such as SM490B, and low-alloy steels such as SNCM435 when exposed to high-pressure hydrogen gas. In materials such as the SM490B and SNCM435 steels, the fatigue crack propagation rate varies with the hydrogen gas pressure. However, in hydrogen gas, the fatigue crack growth (FCG) rate was up to 30 times faster than that in air, particularly when the material’s tensile strength (σ_B_) was below 900 MPa [16], whereas in materials such as the SUS304 and SUS316L steels, the rate accelerates by approximately two to five times [17]. Therefore, estimating fatigue crack propagation using these acceleration factors is crucial for ensuring accurate assessments of materials. For instance, the fatigue crack growth rate in an uncharged specimen, which is 1 × 10^−7^ m/cycles, accelerates to 3 × 10^−6^ m/cycles in the presence of hydrogen when Δ*K* = 30 MPa·m^1/2^. This is observed for SCM435 steel, which has a tensile strength (*s*_B_) of 824 MPa [16]. Here, Δ*K* is the stress intensity factor, which is determined by the stress condition, crack length, and shape of the object.

In facilities such as hydrogen stations, periodic nondestructive testing is conducted manually to ensure safety by examining the presence of cracks originating from internal components such as pipelines, tanks, and valves. However, the current practice is not performed frequently, partly because of the initial use of safe materials, such as JIS-SUS316L.

Eddy current testing is widely used for stainless steel such as SUS316L because of its non-magnetic properties, which allow for the effective detection of surface and near-surface defects [32,40,41,42]. Takeda et al. [32] revealed that the fusion of eddy current testing (ECT) and ultrasonic testing (UT) has been investigated as an effective tool to clarify the hydrogen embrittlement mechanism of austenitic stainless steels. In contrast, carbon steels are less suitable for eddy current testing because their magnetic properties interfere with the detection process. Therefore, limited studies have been conducted on carbon steels. Although replacing hydrogen infrastructure components, such as pipelines, with low-alloy steels or carbon steels is preferable, a guideline is yet to be established on the frequency of nondestructive testing. Additionally, as demonstrated by NASA, the use of “severely embrittled” or “extremely embrittled” materials increases the risk of hydrogen embrittlement, necessitating frequent inspections. This phenomenon could result in higher labor costs and counteract the intended safety improvements.

This study focused on exploring the feasibility of using carbon steel for hydrogen infrastructure by investigating its fatigue crack propagation characteristics under hydrogen-precharged conditions. By using nondestructive testing techniques such as eddy current testing, we focused on understanding the behavior of carbon steel under hydrogen exposure and developing guidelines for its safe application. In this study, a nondestructive testing technique was devised using external sources for the early detection of cracks in pipes. Currently, nondestructive testing techniques, such as eddy current and hammering testing, have been used. To conduct crack-detection experiments on pipelines, we simulated the hydrogen pipelines used in actual hydrogen stations and created experimental pipes with artificially induced cracks. In hydrogen station pipelines, high-pressure hydrogen is used to fill hydrogen tanks for hydrogen FCVs. Although replicating such conditions in the laboratory would involve conducting pressure fatigue tests with high-pressure hydrogen gas, attaining these conditions requires expensive experimental facilities. Therefore, in this experiment, we conducted internal pressure simulation fatigue tests to achieve a crack pattern like that of the pressure fatigue tests, prepared pipes with internal cracks via a cyclic compression fatigue test, and performed nondestructive testing experiments.

## 2. Experimental Procedure

STKN steel was used as the test material. For the internal pressure simulation fatigue test, specimens with an outer diameter of 14.0 mm, inner diameter of 6.0 mm, and overall length of 300 mm were prepared.

**Figure 1:**
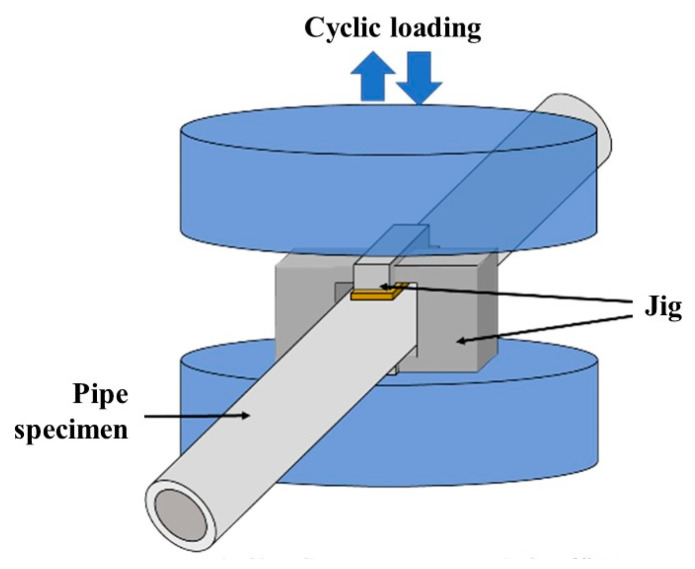
Illustration of quasi-cyclic internal pressure test. A 0.6 mm thick copper plate is interposed to prevent the formation of deep indentations at the specimen surface. Specimens with an outer diameter of 14.0 mm, inner diameter of 6.0 mm, and overall length of 300 mm were prepared. The maximum loads were 20 and 40 kN.

**Figure 2:**
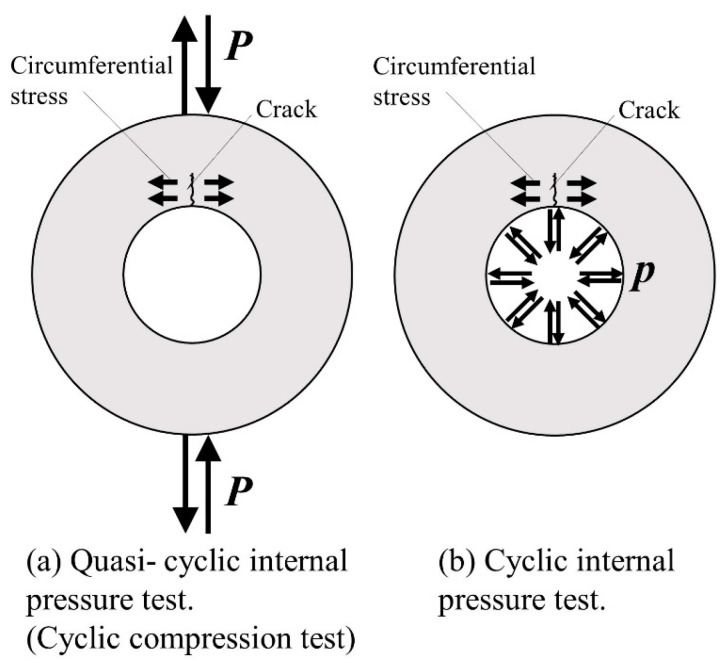
Two cases of the stress state and crack model diagrams.

Figure 1 depicts a schematic of the experimental setup for the quasi-cyclic internal pressure test. A Shimadzu UN500 kN universal testing machine (Shimadzu Corporation, Kyoto, Japan) and a Shimadzu Servo-Pulser fatigue testing machine (Shimadzu Corporation, Kyoto, Japan) were used in the study. In pressure-cycle fatigue testing, cracks propagate radially because of the circumferential stress generated in the inner radius of the pipe. In this study, we used an internal pressure simulation fatigue test method, in which repeated compressive loads were applied from the top and bottom to obtain cracks like those observed in cyclic pressure fatigue tests. Figure 2 depicts the stress state and crack model diagrams for internal pressure applied to the pipe and cyclic compressive loads acting from the top and bottom. As illustrated in Figure 2, in both scenarios, circumferential stress occurred in the inner radius portion of the specimen and cracks propagated radially (as documented in reference by Murakami et al. [18,38]).

The universal testing machine allows testing for a maximum load of 40 kN, and the testing speed was 75 kN/s. For tests conducted on the Shimadzu Servo-Pulser, the maximum load was 20 kN, with a frequency of 10 Hz for the uncharged specimens and 1 Hz for the hydrogen-precharged specimens. Compressive loads were applied to the specimens through blocks of quenched material measuring 20 and 10 mm in length and width, respectively. The stress concentration at the edge of the contact area was alleviated by performing R processing at the ends of the blocks and interposing a 0.6 mm thick copper plate. This approach aims to prevent the formation of deep indentations on the specimen surface as much as possible.

The specimens were subjected to repetitive compressive loading at 40 kN up to *N* = 16,000, followed by hydrogen charging and additional repetitive compressive loading at 40 kN, which resulted in a fracture at *N* = 113. This process was implemented to conduct experiments with as much hydrogen remaining in the test specimen as possible because hydrogen escapes from the specimen over time.

### 2.1. Hydrogen Charging Method and Measurement of Hydrogen Ingress

The immersion method was used to perform hydrogen charging. Test specimens were immersed in a 40 °C, 20% mass solution of ammonium thiocyanate for 72 h, as evidenced by the saturation of hydrogen ingress in the specimens during this immersion period. Consequently, the optimal immersion time was 72 h. To prevent solution intrusion into the internal piping of the test specimens, a covering was applied to both ends of the piping test specimens, because cracks are more likely to occur because of corrosion effects when solution intrusion and internal corrosion occur. Hydrogen charging was subsequently performed. The hydrogen-precharged specimens were stored in a −80 °C freezer until the testing time to prevent hydrogen release into the atmosphere. Prior to testing, the specimens were thoroughly immersed in ethanol and allowed to return to room temperature. A gas chromatography–thermal desorption analyzer (TDA) (J-Science Lab Co., Ltd., Kyoto, Japan) was used to perform hydrogen ingress measurements. To investigate the hydrogen ingress, two methods were used: (1) immersing the entire pipe and (2) charging the specimens with the ends of the internal pipe covered. Additionally, the specimens were immersed for 24, 48, and 72 h, and the hydrogen ingress in the internal piping was investigated to study the hydrogen ingress characteristics.

### 2.2. Nondestructive Inspection System

**Figure 3:**
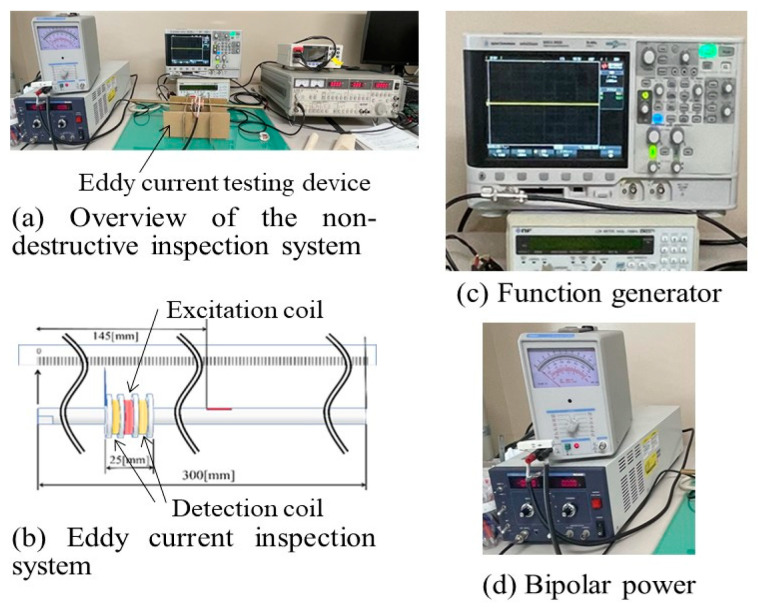
Eddy current testing device.

Figure 3 depicts the eddy current testing device. Figure 3a shows an overview of the nondestructive inspection system and Figure 3b illustrates the eddy current inspection system. In the eddy current inspection system, a self-comparison method was used. The excitation coil was positioned between detection coils in the experimental setup. The steel pipe test specimen for inspection was placed at the center of the coils. The frequency of the excitation coil was varied at 1, 3, and 5 kHz to conduct the experiments and compare the inspection results based on frequency differences. The current was set at 0.1 A, and the inspection involved moving across the range of 50 to 250 mm from the left end of the 300 mm total length of the test specimen when recording the frequency of the excitation coil at each point. Figure 3c depicts the function generator (DSO-X 2002A, Agilent Technologies, Tokyo, Japan) used to convert the current generated in the eddy current inspection system into AC and feed it into the excitation coil. Figure 3d depicts the bipolar power supply (PBA20-12, TEXIO, Yokohama, Japan) used as the power source for the eddy current inspection system. A LOCK-in amplifier (LI5640, NF Corporation, Yokohama, Japan) was used to apply a sine wave reference signal to the input signal and, by passing it through a low-pass filter, only the signal synchronized with the reference signal was detected.

**Figure 4:**
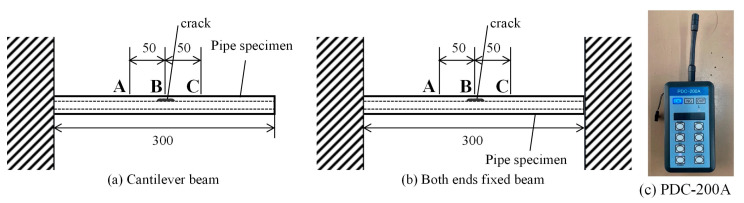
Impact inspection system.

Figure 4 illustrates the impact inspection system. Figure 4a depicts the cantilever beam and Figure 4b reveals both ends of the fixed beam. Figure 4a,b depicts the striking positions in the hammering tests. The test was performed under the following five conditions, namely, the crack-free test specimens, uncharged-material test specimens (*N* = 120,000 and 450,000), and hydrogen-precharged-material test specimens (*N* = 5000 and 50,000). The experimental procedure involved striking points A to C with a percussion rod 30 times in the case of Figure 4a and 20 times in the case of Figure 4b; a PDC-200A system (Kyuken Co., Ltd., Saga, Japan) was used to detect the sound pressure (see Figure 4c).

The pipes were subjected to repeated compressive loading with a maximum load of 20 kN. After repeated compressive loading, each test specimen was held at approximately 400 °C for 3 h, oxidized, and subsequently fractured after being immersed in liquid nitrogen. This phenomenon allowed the confirmation of areas within the pipes in which crack propagation occurred during fatigue testing.

### 2.3. Finite Element Method (FEM) Stress Analysis

#### Analysis of Changes in the Distance between Loading Points

**Figure 5:**
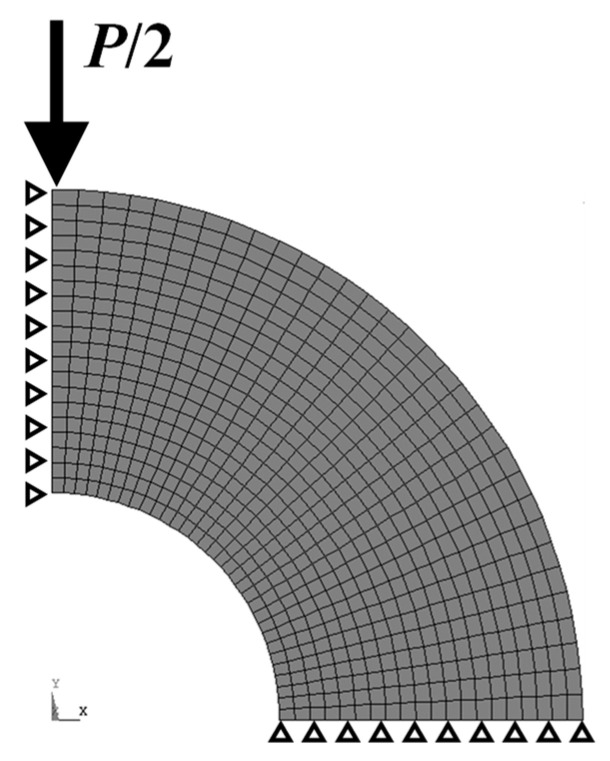
FEM 1/4 model of the compressed pipe.

Figure 5 illustrates the model diagram for finite element analysis (FEM). The elements consisted of eight-node quadrilateral elements and formed a 1/4 model. By applying a load to the point shouwn in Figure 5, circumferential stress was exerted at the inner radius point B, which resulted in the initiation and propagation of radial cracks. During this process, the displacement at load point A increased as the cracks progressed. To verify this phenomenon, an FEM analysis was conducted. Displacement at point A was determined for the crack lengths (*a*) at point B corresponding to 0, 1/5, 2/5, …, 5/5 of the thickness (*t*). Stress at point B was consistent with the ideal value [38] within an error of 5%.

## 3. Experimental Results

### 3.1. Results of Hydrogen Measurement

**Figure 6:**
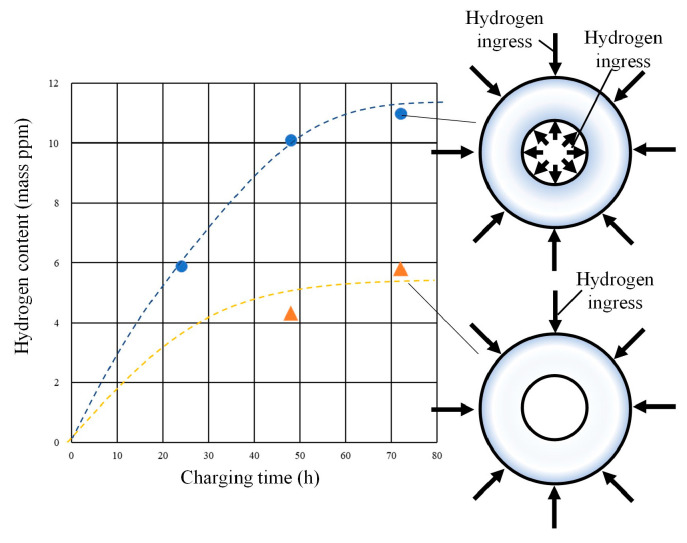
Relationship between hydrogen charging time and hydrogen content.

Figure 6 depicts the relationship between the hydrogen charging time and hydrogen ingress content. The results are presented for two scenarios: one scenario in which hydrogen can ingress from both the inner and outer surfaces of the piping and another scenario in which hydrogen can ingress only from the outer surface of the piping. In cases in which hydrogen can ingress from both sides, the hydrogen volume increased considerably for up to 48 h, after which a minimal increase was observed in the hydrogen concentration, reaching 10 mass ppm and beyond. This phenomenon indicates that the saturated hydrogen concentration in this study was approximately 10 mass ppm. However, when a cover was applied to both ends of the piping, the cover prevented contact with the aqueous solution at the inner radius and the saturated hydrogen concentration was approximately 6 ppm. This phenomenon could be attributed to the fact that the inner radius portion was exposed to the atmosphere, which allowed hydrogen to penetrate from the outside and be released from the inside of the piping. Even in this condition, the hydrogen ingress reached saturation within 72 h. Based on this result, the hydrogen charging time was determined to be 72 h.

### 3.2. Crack Growth Property

**Figure 7:**
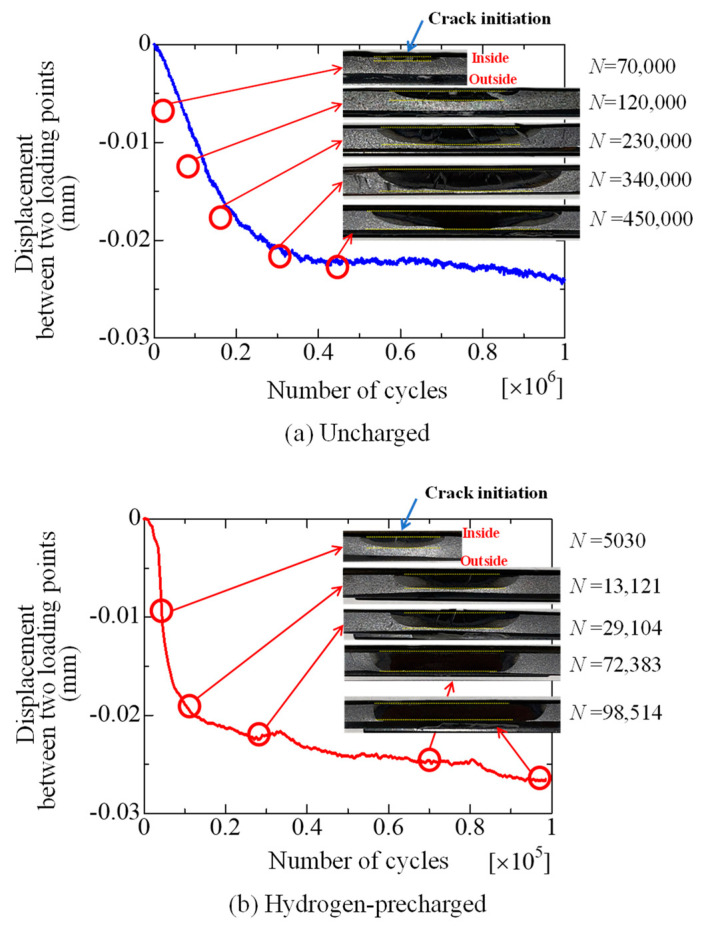
Relationship between number of cycles and displacement between two loading points (*P*_max_ = 20 kN). This figure illustrates how, as the number of cycles increases, the crack that forms inside the pipe gradually extends, leading to a larger amount of displacement between the loading points. In (**a**), the relationship for uncharged material is shown, including photographs of crack progression at *N* values of 70,000, 120,000, 230,000, 340,000, and so on. On the other hand, (**b**) shows this relationship for hydrogen-precharged material, including photographs of crack progression at *N* values of 5030, 13,121, 29,104, and so on.

**Figure 8:**
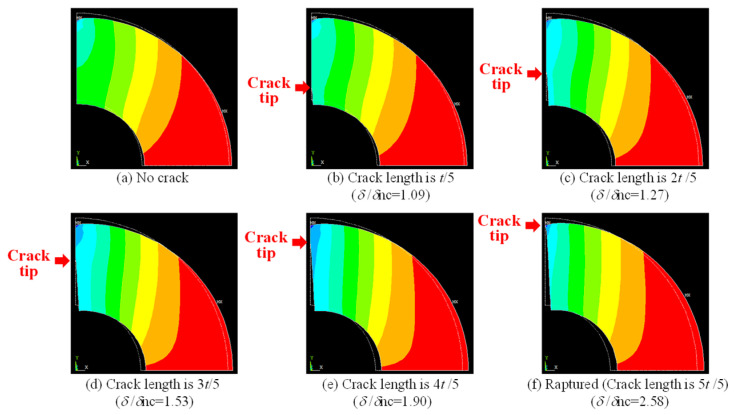
Results of the FEM analysis for the compression-loaded 1/4 pipe model at each crack growth length. *d* represents the change in distance between loading points in each case, while *d*nc denotes the change in distance between loading points in case of (**a**) No crack. The white dotted line represents the shape before deformation. The color gradient indicates displacement, with blue representing larger displacement and red representing minimal displacement. (**b**) Crack length is *t*/5, (**c**) Crack length is 2*t*/5, (**d**) Crack length is 3*t*/5, (**e**) Crack length is 4*t*/5, (**f**) Ruptured (Crack length is 5*t*/5).

Figure 7 displays a graph depicting the relationship between the number of cycles and displacement between the two loading points for the quasi-cyclic pressure test. This figure illustrates how, as the number of cycles increases, the crack that forms inside the pipe gradually extends, leading to a larger amount of displacement between the loading points. In Figure 7a, the relationship for uncharged material is shown, including photographs of the crack progression at *N* values of 70,000, 120,000, 230,000, 340,000, and so on. On the other hand, Figure 7b shows this relationship for a hydrogen precharged material, including photographs of the crack progression at *N* values of 5030, 13,121, 29,104, and so on. Figure 7 reveals that the displacement of the hydrogen-precharged specimen was greater than that of the uncharged specimen after a lower number of cycles, despite the load conditions being constant. Figure 8 reveals the FEM analysis results for the change in the displacement of the loading point with crack propagation. Here, *d* represents the change in distance between loading points in each case, whereas *d*nc denotes the change in the distance between loading points in case of (a) No crack. As indicated in Figure 8, the displacement *d/d*nc increased with the crack length. Considering the results in Figure 7 and Figure 8, hydrogen accelerated the increase in the crack propagation ratio. Specifically, Figure 7 demonstrates that the displacement of the uncharged specimen decreased by 0.02 after 2 × 10^5^ cycles, whereas the hydrogen-precharged specimen exhibited the same displacement decrease after only 2 × 10^4^ cycles. This phenomenon indicates that the displacement at the load point is decreasing, meaning that internal cracks are propagating. Therefore, the hydrogen-precharged specimen experiences crack propagation approximately ten times faster than that of the uncharged specimen. Figure 7 also reveals the fractured specimens for each loading cycle. The crack propagation pattern reveals that the crack extends up to approximately the outer surface when the displacement is approximately −0.02 mm. After cycling, the displacement remained almost constant. The cracks that started from the inner surface of the pipe were accelerated by approximately 10 times until they reached the outer surface.

### 3.3. Fracture Morphologies

**Figure 9:**
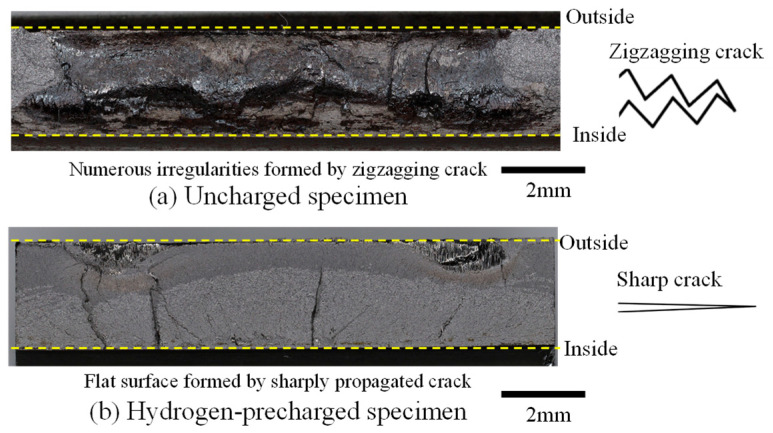
Fracture surface morphologies and crack propagation illustration for uncharged and hydrogen-precharged specimens (*P*_max_ = 40 kN).

Figure 9 presents the stereomicroscopic photographs of the fracture surfaces of the fractured specimens under the load condition of *P*_max_ = 40 kN. Figure 9a shows the un-charged specimen, whereas Figure 9b shows the hydrogen-precharged specimen. In Figure 9a, the fracture surface of the uncharged specimen exhibited numerous irregularities along the convex or concave surface of the crack, which indicates that the cracks originated internally within the pipe and propagated outward in a zigzag pattern. By contrast, the hydrogen-precharged specimen in Figure 9b displays a flat surface with a few prominent bumps and dips.

Kanezaki et al. [10] demonstrated crack growth in the austenitic stainless steels SUS304 and SUS316L. In this study, austenite transformed into martensite at the crack tip and hydrogen diffusion was concentrated at the crack tip through crystals undergoing martensitic transformation, which indicated that hydrogen enhanced localized plasticity at the crack tip and linear crack growth. Yoshikawa et al. observed distinct crack growth behavior in a hydrogen gas environment for carbon steel JIS-SM490B [36,37]. They noted that the crack tip remains sharp and does not blunt, resulting in an increased spacing of striations during fatigue crack propagation due to the presence of hydrogen. The fracture surface morphology results obtained in this study were consistent with the findings reported in previous studies.

### 3.4. Nondestructive Flaw Detection

**Figure 10:**
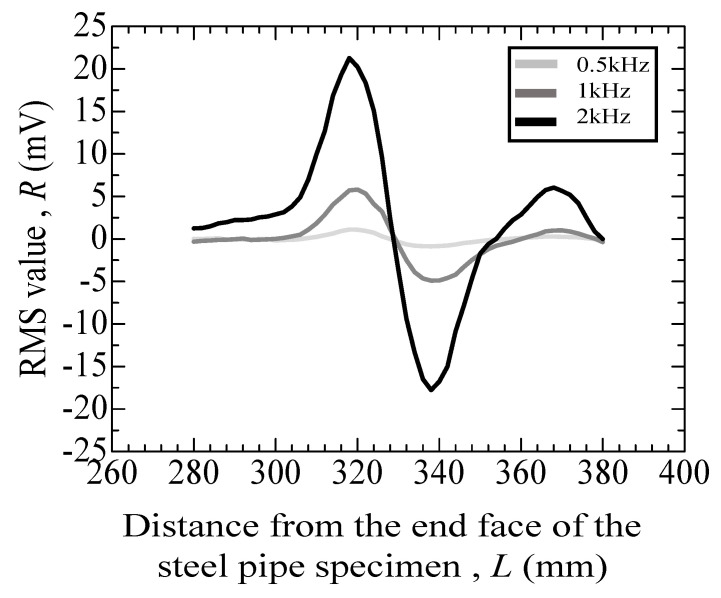
Eddy current testing result of a standard specimen with indentations of 1.0 mm in diameter and 0.1 mm in depth.

Figure 10 displays the results of a standard specimen with indentations of 1.0 mm in diameter and 0.1 mm in depth. The graph from Figure 10 exhibits a sinusoidal wave cycle. Higher frequencies correspond to lower RMS values of the voltage because of the concentration of eddy currents in the conductor at higher frequencies, which increases sensitivity.

**Figure 11:**
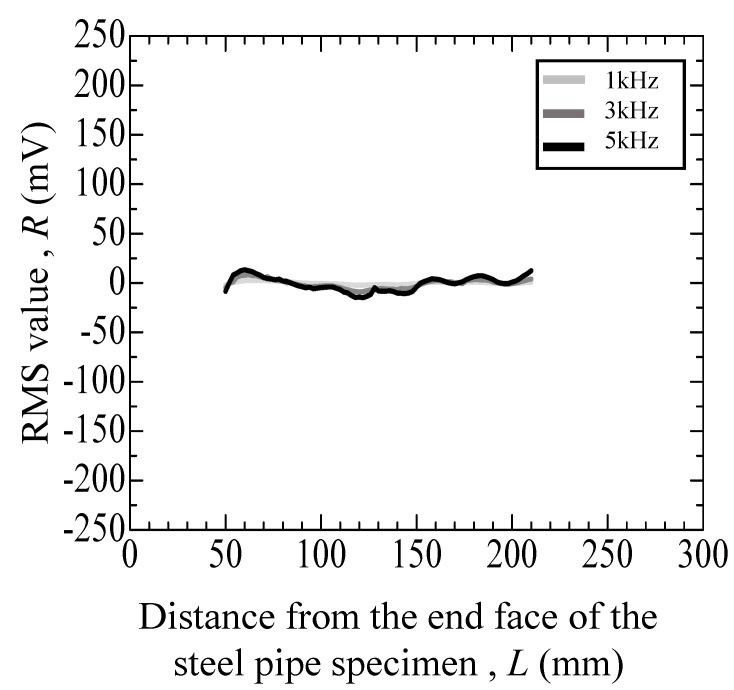
Eddy current testing result of a pipe specimen without an indent or crack.

**Figure 12:**
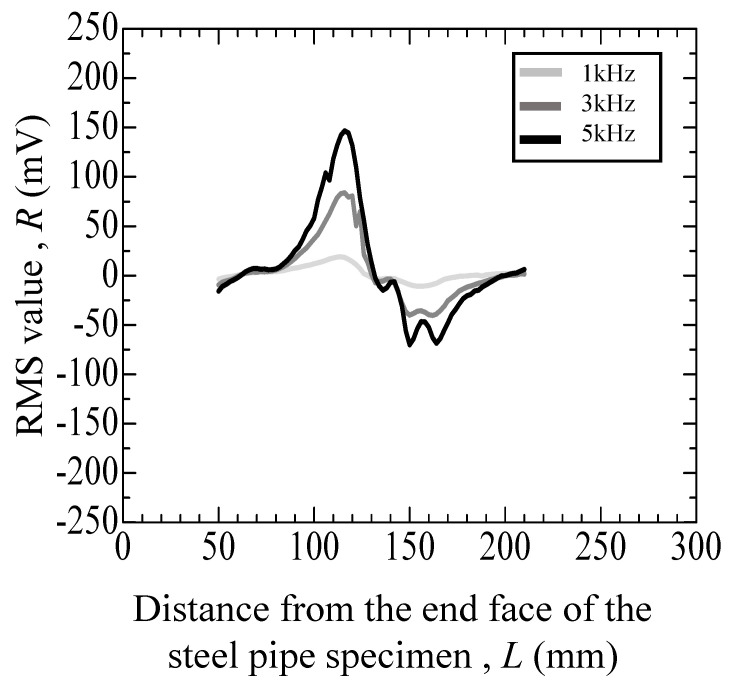
Eddy current testing result of a pipe specimen with an indent (depth: 100 μm) and without cracks.

**Figure 13:**
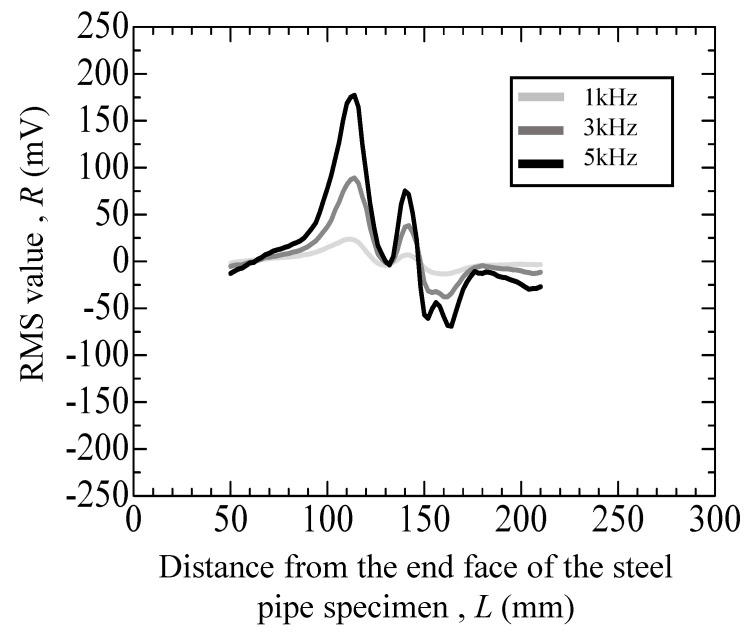
Eddy current testing result of the uncharged specimen with an indent (depth: 200 μm) and with a crack.

**Figure 14:**
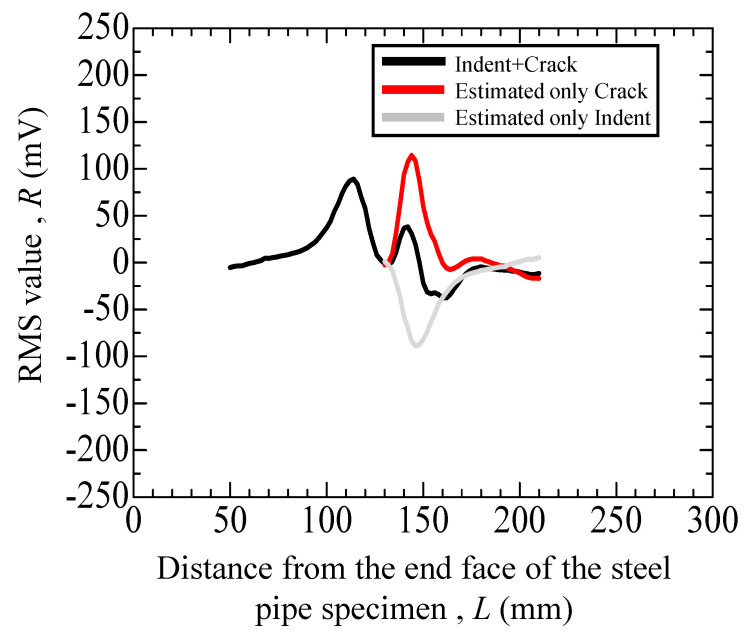
Eddy current testing result (3 kHz) of the uncharged specimen with an indent (depth: 200 μm) and with a crack.

**Figure 15:**
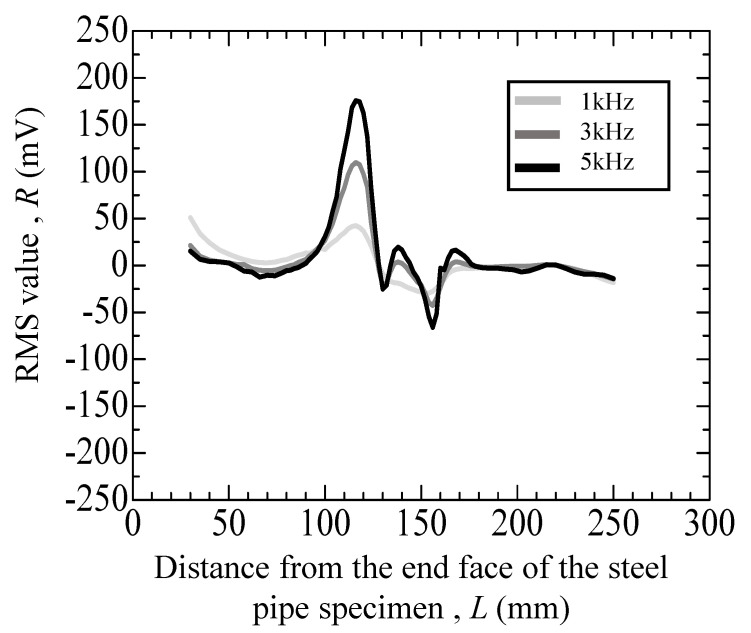
Eddy current testing result of the hydrogen-precharged specimen with an indent (depth: 200 μm) and with a crack.

Figure 11, Figure 12, Figure 13, Figure 14 and Figure 15 depict the variation in the root mean square (RMS) values of the voltage acquired from the detection coil during the eddy current testing of the specimen. The vertical axis represents the RMS value *R* (mV), and the horizontal axis represents distance *L* (mm) from the end face of the steel pipe specimen.

Figure 11 reveals the test results for the specimen that did not undergo the internal pressure-simulated fatigue test. As depicted in Figure 11, the error for the specimens without cracks is as follows: at 1 kHz, it ranges from −2.27 mV to +0.75 mV; at 3 kHz, from −8.68 to +3.43 mV; and at 5 kHz, from −14.87 to +6.42 mV. Table 1 summarizes the results of the eddy current testing results. Figure 12 reveals the results of a specimen that incurred an indent of approximately 100 µm. As depicted in Figure 12, numerous peaks are observed at 100 to 200 mm for the specimen with a large indent on the surface. Additionally, the peaks become higher with the increase in the frequency, while the positions of peaks remain consistent.

Figure 13 displays the results of repeated compression fatigue testing at a maximum load of 40 kN on a specimen with a crack originating from the inner surface penetrating the outer surface. The indent size was approximately 200 µm. In Figure 13, a large peak appears in the range of 100–200 mm, which is caused by an indent, as depicted in Figure 12. In addition, a peak was observed near 145 mm in Figure 13. This peak near 145 mm is absent in Figure 12, as the specimen did not exhibit cracks. This difference indicates that this peak is indicative of cracks. The peak heights at 145 mm for the uncharged specimens are as follows: at 1 kHz, 7.15 mV; at 3 kHz, 38.14 mV; and at 5 kHz, 71.4 mV. The positions of the peaks remain consistent across frequencies, whereas their amplitudes increase with higher frequencies.

Figure 13 is the result of the overlapping graphs because of indentation and cracking. Therefore, the peaks were separated considering the 120 mm peak caused by indentation as a peak in which the ups and downs were reversed again like a sine wave. The red curve in Figure 14 depicts the results of the estimated graph for only crack after the peak separation at 3 kHz. As a result of peak separation, the peak magnitude due to the crack was 114 mV, which is higher than the peak caused by indentation.

Figure 15 presents the test results of a fractured specimen composed of hydrogen-precharged materials. The specimen exhibited a prominent peak in the range 100–130 mm, along with several smaller peaks in the range 130–170 mm. The peaks between 100 and 130 mm were attributed to indents, whereas those between 130 and 170 mm were a combination of indent and crack peaks. The peak heights at 140 mm for the hydrogen-precharged specimens are as follows: at 1 kHz, −20.27 mV; at 3 kHz, 2.35 mV; and at 5 kHz, 16.66 mV. Similar to Figure 13 for an uncharged specimen, the positions of the peaks remained consistent across frequencies, whereas their amplitudes increased with higher frequencies. However, the heights of the peaks appear smaller compared to those of the uncharged specimens. This phenomenon indicates that the eddy current testing of hydrogen-precharged specimens has some difficulty.

**Figure 16:**
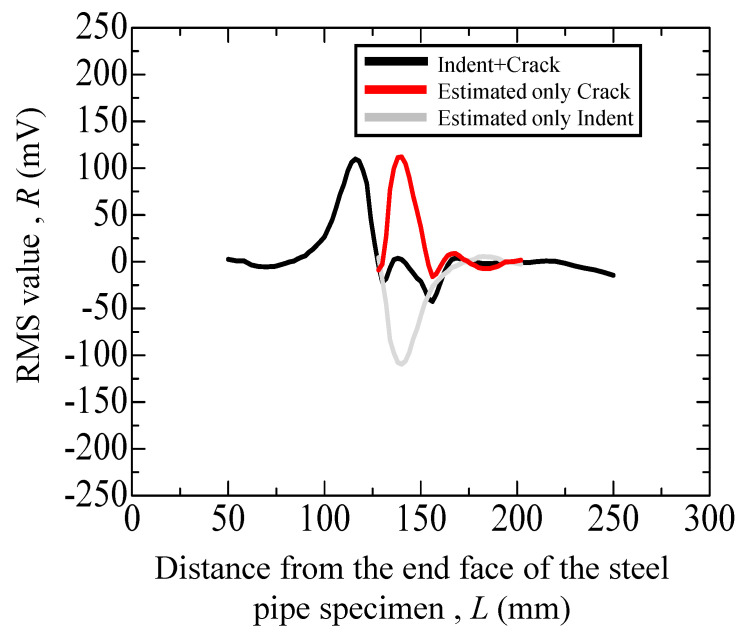
Eddy current testing result (3 kHz) of the hydrogen-precharged specimen with an indent (depth: 200 μm) and with a crack.

Figure 16 presents the separated graph at 3 kHz, analogous to those depicted in Figure 14. As indicated, the peak value reached 111 mV, which is nearly the same value observed for the uncharged specimen in Figure 14. Consequently, this method could effectively detect cracks in hydrogen-precharged materials with minimal surface irregularities, as depicted in Figure 9. However, when a crack propagates in a zigzag pattern, gaps are more likely to form in those areas, causing disturbances in the eddy current flow. By contrast, if the crack propagates in a straight line via the hydrogen effect, then the crack tends to remain less open, rendering eddy current flow difficult. This difference in crack propagation patterns likely leads to variations in detection sensitivity.

Eddy current testing was conducted when the crack extended to the halfway point. Therefore, the results did not reveal any significant peaks, indicating that depth probing is difficult. The eddy current method is more effective for detecting external cracks. Another feature of the eddy current method is that the method is susceptible to shape changes. Therefore, hammering testing methods were investigated.

## 4. Hammering Test

**Figure 17:**
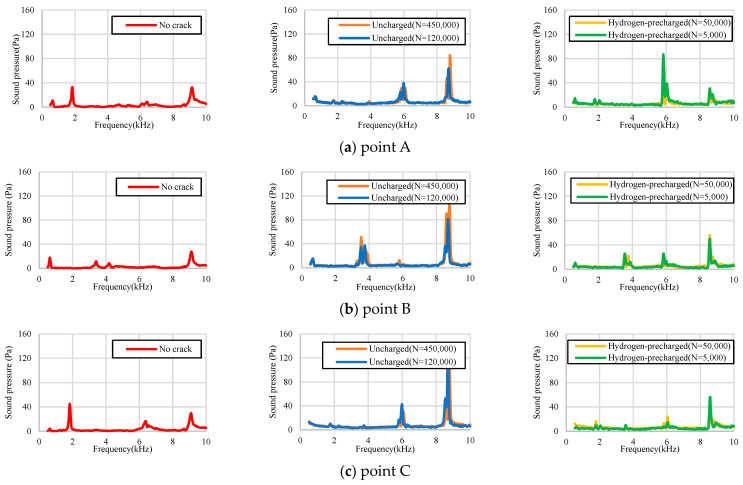
Result of the hammering testing conducted on a cantilever beam. (**a**) Striking point A, (**b**) Striking point B, (**c**) Striking point C. Refer to Figure 4 for the striking point A–C.

**Figure 18:**
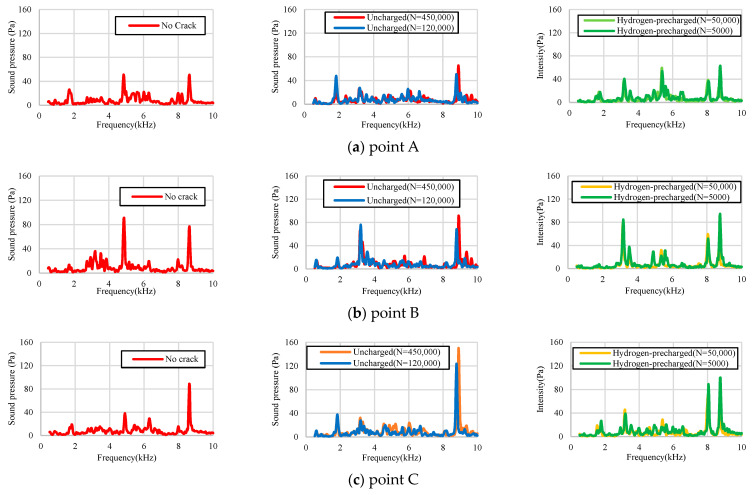
Results of the hammering testing for both ends supported beams. (**a**) Striking point A, (**b**) Striking point B, (**c**) Striking point C. Refer to Figure 4 for the striking point A–C.

Figure 17 reveals the results of the hammering tests conducted on the cantilever beam, as depicted in Figure 15. Figure 17a reveals the results for the pipe without a crack, Figure 17b depicts the results for the uncharged test specimen, and Figure 17c details the results for the hydrogen-precharged test specimen. A common characteristic observed across (a) to (c) is the manifestation of peaks within the 9 kHz frequency range. This phenomenon could be attributed to the inherent material properties of the specimens. Furthermore, both the uncharged and hydrogen-precharged test specimens exhibited a peak at 3.5 kHz, a feature that is not discernible in the crack-free material. This result could be attributed to the presence of cracks, which were observed in the frequency range of 3.5 kHz. The major difference between the uncharged and hydrogen-precharged specimens was that the hydrogen-precharged specimens exhibited a peak at 6 kHz. This result can be explained as follows. As presented in Figure 9, the cracks in the hydrogen-precharged specimens were sharp and straight, and the cracks closed when load was not applied. However, the uncharged specimen had a large opening and zigzagging crack, which revealed that a space was created at the crack surface when load was not applied. This difference in the sound wave path could be attributed to the differences in the frequency spectra in Figure 18b,c.

Figure 18 depicts the results of the hammering testing for both ends of the supported beams.

The uncharged and hydrogen-precharged specimens exhibit a prominent peak at 3.19 kHz at crack initiation point B. This peak was not observed for the uncracked specimen. This phenomenon indicates that the 3.19 kHz peak is a peak that is unique to the cracked material. The results were similar for both uncharged and hydrogen-precharged specimens, as well as for the specimens with through-thickness cracks and half-thickness crack extensions. Although the frequency at which the peak appears can vary depending on the geometry of the structure and the anchoring method, a frequency was detected in both the uncharged and hydrogen-precharged specimens. Furthermore, this result is possible even when the crack has propagated only halfway along the thickness direction. Figure 17 and Figure 18 reveal that the major difference between the uncharged and hydrogen-precharged specimens is that only the hydrogen-precharged specimen exhibited a peak at 8.05 kHz. Furthermore, a broad peak was observed at 5–6 Hz. However, the peak height was low. This phenomenon indicates that the frequencies at which the peaks appeared changed because of the crack geometry between the uncharged and hydrogen-precharged specimens. Thus, the hydrogen-precharged specimen exhibits the same characteristics as the uncracked specimen because the crack was sharply propagated and the top and bottom surfaces of the crack were in contact with each other. This phenomenon could be attributed to the similarity between the hydrogen-precharged specimen, with the crack extending halfway up the thickness, and the crack-free specimen, with a peak at 5 Hz. This result was not observed in the uncharged specimens with half-thickness crack propagation. The uncharged specimens exhibited a wide plastic zone at the crack tip, which could be attributed to a large crack opening, as previously shown. The hydrogen-precharged specimens with cracks extending to the penetration point also exhibited large crack elongation and a large crack aperture, which could be attributed due to a reduction in the area of contact between the top and bottom surfaces of the crack.

In the future, nondestructive testing can be considerably enhanced by the integration of artificial intelligence (AI) and the Internet of Things (IoT). These advancements can enable remote crack detection and monitoring without the necessity of human intervention. Automated systems equipped with AI algorithms can analyze data in real time, providing immediate feedback and predictive maintenance insights. IoT devices will facilitate seamless data communication between inspection sites and central analysis hubs, ensuring continuous and accurate monitoring of pipeline integrity. This convergence of technologies can revolutionize the field, rending inspections efficient, reliable, and cost effective.

## 5. Conclusions

The fatigue test was performed by applying cyclic compressive loads above and below STKN steel piping with an inner diameter of 8 mm and outer diameter of 14 mm to induce crack propagation from the inner surface for both uncharged and hydrogen-precharged specimens. Hydrogen precharging was performed by immersing the piping specimens in a 40 °C, 20 mass% ammonium thiocyanate solution. Subsequently, internal cracks were probed using eddy current and hammering testing. The results obtained were as follows:

1. When the piping specimens were immersed in a 20 wt% ammonium thiocyanate solution at 40 °C, the hydrogen content reached the saturation value after 72 h. Thus, the optimal immersion time was set to 72 h.
2. The crack growth rate of the hydrogen-precharged specimen was accelerated by hydrogen compared with that of the uncharged specimen, and the cracks that started from the inner surface of the pipe were accelerated by approximately 10 times until they reached the outer surface.
3. The fracture surface morphology of the cracks exhibited a flat fracture surface in the hydrogen-precharged material, whereas the morphology was convex or concave in the uncharged material.
4. The results of eddy current and hammering tests revealed differences in the presence and absence of large cracks in the uncharged and hydrogen-precharged materials. In the eddy current test, clear results were not obtained for the half-cracked material, whereas in the hammering test, the results were similar to those obtained for the fractured and half-cracked materials. No significant differences were observed between the uncharged and hydrogen-precharged materials.

## Figures and Tables

**Table 1:** Summarizing the eddy current testing results.

Load *P* (kN)	Figure	Hydrogen Precharging	Number of Loading Cycles	Crack and Dent	Peaks of RMS Value for Crack Detection
-	Figure 11	Uncharged	-	without crack and indent	No peak
40	Figure 12	Uncharged	*N* = 6000	without crack and with indent	No peak
40	Figure 13	Uncharged	*N* = 20,498	with crack and indent	+75 (mV)
40	Figure 15	Hydrogen precharged after cyclic loading of uncharged *N* = 16,000	*N* = 113	with crack and indent	+25 (mV)

## Data Availability

The data presented in this study are available on request from the corresponding author. The data are not publicly available due to privacy restrictions.
